# Recognition Element-Based Strategies for Rapid Detection of Foodborne Pathogens: Recent Progress and Perspectives

**DOI:** 10.3390/bios15110717

**Published:** 2025-10-29

**Authors:** Wang Guo, Meifeng Jiang, Yunkai Xie, Hong Xu, Zongbao Sun

**Affiliations:** 1 School of Food & Biological Engineering, Jiangsu University, Zhenjiang 212013, Chinaxyk21987@163.com (Y.X.); 2 Zhenjiang Center for Disease Control and Prevention, Zhenjiang 212013, China; jszjxh1@163.com

**Keywords:** foodborne pathogens, rapid detection, recognition elements, CRISPR, microfluidics

## Abstract

The detection of foodborne pathogens is of great significance for safeguarding food safety and public health. In recent years, rapid detection technologies based on diverse recognition elements have advanced considerably, driven by progress in molecular biology, materials science, and information technology. This review takes recognition elements as the central theme and systematically outlines the mechanisms and research progress of antibodies, nucleic acid aptamers, nucleic acid amplification techniques, CRISPR/Cas systems, molecular imprinting technology, peptides, and small-molecule receptors in foodborne pathogen detection, while comparing their performance in terms of specificity, sensitivity, stability, and applicability. In addition, this review further elaborates on the developmental trends of detection platforms, including multi-target and multimodal integration, microfluidics combined with portable point-of-care testing (POCT) systems, and intelligent terminals empowered by artificial intelligence algorithms. These trends provide new perspectives for improving detection systems in terms of throughput, portability, and intelligence. Overall, this review aims to serve as a comprehensive reference for the development of rapid, accurate, and intelligent detection systems for foodborne pathogens.

## 1. Introduction

Foodborne pathogens are among the most critical microbial threats to global food safety, encompassing *Staphylococcus aureus* (*S. aureus*), *Salmonella*, *Escherichia coli* (*E. coli*), *Listeria monocytogenes* (*L. monocytogenes*), *Shigella*, and *Campylobacter*, among others [1,2,3,4,5,6]. Once transmitted through contaminated food or water, these pathogens can cause diarrhea, vomiting, fever, and even severe septicemia, posing a serious risk to human health and potentially leading to fatal outcomes. According to the World Health Organization (WHO), an estimated 600 million people worldwide suffer from foodborne illnesses each year, with approximately 420,000 deaths [7]. Beyond the direct public health burden, recurrent outbreaks undermine consumer confidence, disrupt food supply chains and international trade, and inflict substantial economic losses.

The transmission pathways of foodborne pathogens are complex and multifaceted, spanning food production, processing, storage, distribution, and consumption [8]. High-nutrient foods such as dairy products, meat, and seafood provide favorable conditions for rapid microbial proliferation when stringent temperature and hygiene controls are absent [9,10]. Fresh produce can also be contaminated during harvesting, washing, or packaging, while drinking water, utensils, packaging materials, and even human handling may serve as vectors for cross-contamination [11,12]. Compounding these risks, many foodborne pathogens exhibit considerable environmental resilience and growing antibiotic resistance, which limits the effectiveness of conventional treatment strategies [13].

Conventional culture-based methods remain the cornerstone of pathogen detection, offering intuitive and reliable results and long regarded as the “gold standard” [14]. These techniques rely on promoting bacterial growth on selective media, followed by identification based on morphological, metabolic, or biochemical characteristics. However, culture-based detection is inherently time-consuming, often requiring several days to a week, and is therefore incompatible with the rapid response demands of modern food safety management. Furthermore, certain pathogens are slow-growing or even non-culturable under laboratory conditions, which restricts the applicability of this approach. In light of increasingly complex food supply chains and stringent regulatory requirements, there is an urgent need for the development of novel detection technologies that are rapid, sensitive, and adaptable to real-world settings.

Recent advances in molecular biology, materials science, nanotechnology, and artificial intelligence have fueled the emergence of innovative rapid detection strategies. These approaches offer distinct advantages, including high sensitivity, strong specificity, short assay times, and suitability for on-site deployment, making them indispensable tools for food safety surveillance. The continuous evolution of recognition elements—from conventional antibodies to aptamers, nucleic acid amplification methods, CRISPR-Cas systems, and functionalized nanomaterials—has been instrumental in enhancing assay performance [15,16,17,18,19]. Concurrently, the integration of microfluidic platforms and portable analytical devices has enabled the miniaturization, automation, and systematization of detection processes [20]. Furthermore, the incorporation of artificial intelligence (AI) and the Internet of Things (IoT) has propelled the transition toward intelligent and digital detection systems, facilitating real-time monitoring, remote analysis, and large-scale data-driven surveillance [21].

**Figure 1:**
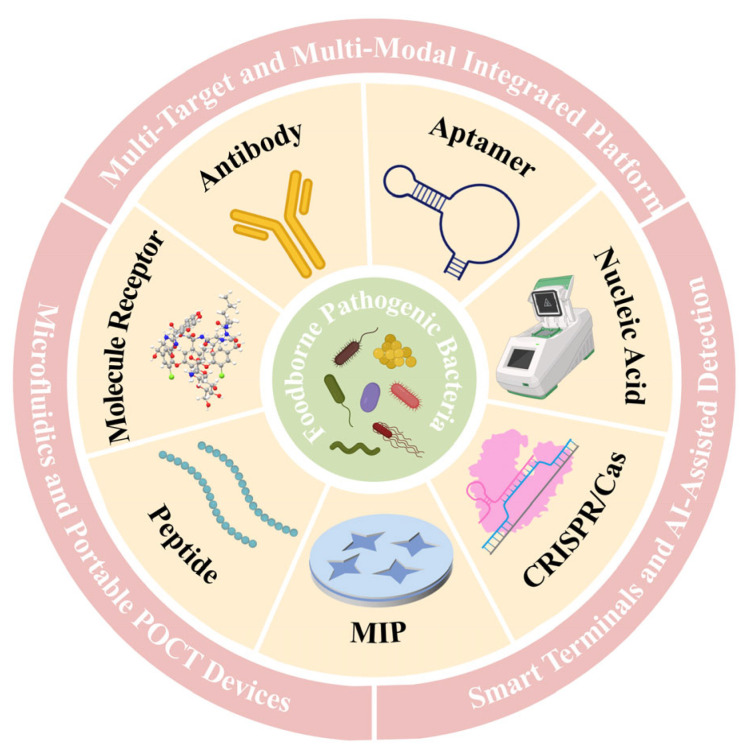
Schematic illustration of recognition element-based rapid detection strategies for foodborne pathogens and their integrated platform.

As illustrated in Figure 1, this review provides a systematic overview of recent advances in rapid detection of foodborne pathogens, organized around recognition elements as the central theme. By analyzing the strengths and limitations of different recognition strategies, this review aims to elucidate the critical challenges that current detection systems face and to highlight future research directions. The goal is to offer theoretical insights and technical references for researchers and regulatory authorities, ultimately advancing the innovation and practical deployment of rapid pathogen detection technologies in food safety management.

## 2. Sample Preparation and Matrix Effects in Food Detection

In recent years, rapid detection technologies for foodborne pathogens have made significant progress and demonstrated broad application prospects in food safety assurance and clinical diagnostics. However, the complexity of food samples remains a major barrier to the reliability of detection outcomes. Target bacteria often exist at extremely low concentrations and are masked by abundant food components and coexisting microorganisms. This not only reduces detection sensitivity but also increases the risk of false-negative results [22]. Consequently, effective sample pretreatment to eliminate interferences and enrich target bacterial populations has become an indispensable step in rapid detection workflows.

Traditionally, enrichment culture has been employed to increase bacterial counts to the threshold required for detection. Nevertheless, this process is time-consuming and poorly suited to damaged or dormant cells, which may fail to regain viability, thereby creating discrepancies between detection results and actual contamination levels [23]. To accelerate the process, a variety of physical and immunological methods have been developed, including filtration, centrifugation, and immunomagnetic separation [24,25]. Filtration leverages differences in pore size to remove impurities and concentrate bacteria, offering speed and cost-effectiveness but limited efficiency in high-solid or low-bacterial-load samples. Centrifugation exploits density and size differences to sediment bacteria under high-speed rotation, applicable across diverse matrices but lacking selectivity. In contrast, immunomagnetic separation integrates the specific recognition capacity of antibodies with the manipulability of magnetic particles, enabling rapid capture and separation of target pathogens even in complex environments, making it particularly advantageous for low-abundance contaminations.

More recently, combined pretreatment strategies have been increasingly explored to overcome the limitations of individual methods. For instance, integrating immunomagnetic separation following impurity removal can simultaneously improve sensitivity and specificity while reducing overall processing time, significantly enhancing the practicality of detection workflows [26]. Although these approaches have been extensively validated under laboratory conditions, their adoption in the food industry remains constrained by costs, operational complexity, and the need for standardization.

Future development should prioritize automated, low-cost, and high-throughput pretreatment solutions, while actively incorporating advanced technologies such as functional nanomaterials and microfluidic platforms to promote the construction of integrated pretreatment–detection systems. Overall, sample pretreatment is not only critical for eliminating matrix interferences and improving detection sensitivity but also lays the foundation for the effective function of recognition elements. How different recognition elements perform in selectively capturing target pathogens from enriched samples will be systematically discussed in the following sections.

## 3. Detection Strategies Driven by Recognition Elements

**Figure 2:**
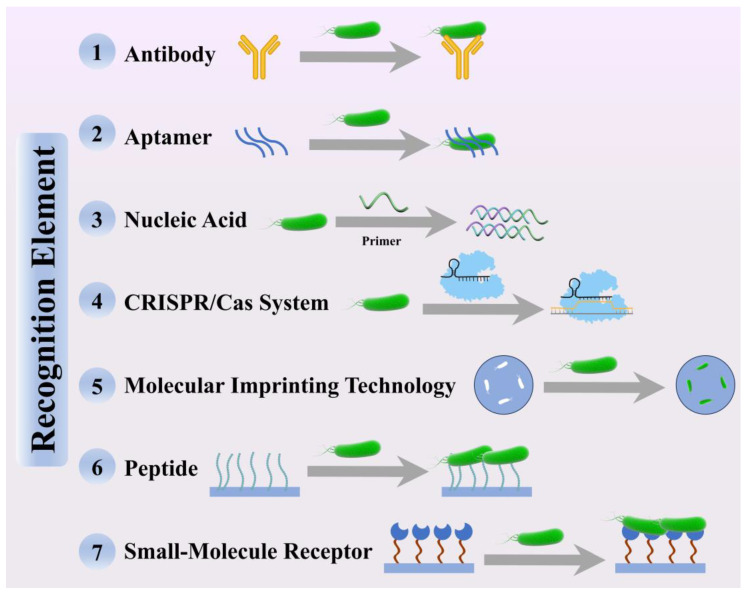
Schematic illustration of the recognition mechanisms of seven representative recognition elements for foodborne pathogen detection.

Recognition elements are the core components of rapid pathogen detection technologies, as they directly determine the specificity, sensitivity, and accuracy of the system. Early detection strategies primarily relied on antibodies and specific nucleic acid fragments, but in recent years, a broader spectrum of recognition tools has emerged, including nucleic acid aptamers, isothermal amplification systems, and CRISPR-Cas platforms (Figure 2). Each recognition element possesses unique mechanisms of action and application scenarios, and researchers typically select or design the most appropriate elements based on the characteristics of the target pathogen and the specific requirements of the assay, thereby enabling efficient target capture and signal transduction.

### 3.1. Antibody

Antibodies represent the most widely applied and well-established class of recognition elements in pathogen detection technologies and are extensively utilized in rapid immunological platforms such as immunochromatography, enzyme-linked immunosorbent assay (ELISA), and immunofluorescence assays [27,28,29,30,31]. Owing to their unique three-dimensional spatial structures, antibodies can bind with high specificity to antigens on the surfaces of foodborne pathogens, such as surface proteins and polysaccharides, thereby enabling efficient capture and detection of the target pathogens [32]. This antigen diversity has facilitated antibody-based assays for *Salmonella* (O-antigen, LPS (lipopolysaccharides)), *E. coli* O157:H7 (intimin, LPS), *S. aureus* (enterotoxins), and *L. monocytogenes* (internalins), illustrating their broad applicability across different classes of bacterial biomarkers [33].

Based on their origin and production methods, antibodies are generally categorized as polyclonal antibodies (pAbs) and monoclonal antibodies (mAbs). Polyclonal antibodies, generated through animal immunization, comprise a heterogeneous mixture that recognizes multiple epitopes of the same antigen, offering broad-spectrum recognition with relatively simple and rapid preparation [34,35]. In contrast, monoclonal antibodies, produced by hybridoma cells, bind specifically to a single epitope, providing superior specificity and batch-to-batch consistency, which makes them more suitable for precise and rapid detection [36]. With advances in genetic engineering and biotechnology, antibody formats have expanded to include nanobodies and recombinant antibodies, which are emerging as highly promising recognition elements in next-generation rapid detection [37,38]. Nanobodies, derived from camelid heavy-chain antibodies, exhibit small molecular size (~15 kDa), high thermal and pH stability, and facile engineering potential. These features make them especially attractive for on-site and resource-limited pathogen testing, with reported binding affinities in the low-nanomolar to-picomolar range, often surpassing those of conventional antibodies [39,40].

Despite the maturity and widespread adoption of antibody-based detection technologies, several inherent limitations persist. The preparation of antibodies, especially monoclonal antibodies, is time-consuming, costly, and technically demanding, often requiring several months to complete [41]. Moreover, antibodies are sensitive to environmental factors such as temperature and pH, which may lead to denaturation or reduced specificity if not properly stored or handled [42]. Another significant drawback is their reliance on specific surface antigens, which renders them susceptible to cross-reactivity or false-negative results when antigenic variations occur in the target pathogens [33]. To address these shortcomings, novel antibody engineering and modification strategies have been proposed in recent years. Techniques such as oriented coupling and antibody–nanomaterial hybrid probes have demonstrated the ability to improve antibody stability, enhance binding specificity, and boost detection sensitivity [43,44].

In summary, antibodies remain the most conventional yet indispensable recognition elements in the field of rapid pathogen detection. Their continued relevance lies in their broad applicability and irreplaceable role, though future research should focus on optimizing production processes, reducing costs, and improving environmental tolerance and sensitivity, thereby driving antibody-based detection technologies toward greater precision, efficiency, portability, and affordability.

### 3.2. Aptamer

Nucleic acid aptamers, generated through the systematic evolution of ligands by exponential enrichment (SELEX), are single-stranded DNA or RNA molecules that exhibit antibody-like specificity and affinity [45,46]. Since their introduction in the 1990s, SELEX and its derivatives (e.g., cell-SELEX, high-throughput SELEX) have enabled the generation of aptamers against a broad spectrum of biomolecules, including bacterial cells, toxins, and nucleic acids [47]. In recent years, they have emerged as promising recognition elements for the rapid detection of foodborne pathogens. Aptamers achieve target recognition, capture, and detection by folding into unique three-dimensional conformations, and primarily rely on van der Waals forces, hydrogen bonding, and other non-covalent interactions with target molecules, thereby binding with high efficiency to specific biomolecules on bacterial cell surfaces, such as proteins, polysaccharides, or other molecular markers. In addition, nucleic acid aptamers can directly hybridize with complementary DNA or RNA sequences in foodborne pathogens, thus enabling highly specific recognition at the genomic level. These diverse mechanisms allow aptamers to bind a wide range of bacterial biomarkers—including surface proteins, LPS, outer membrane proteins, and secreted toxins—with dissociation constants often in the nanomolar to picomolar range [48,49]. Representative examples include aptamers targeting *Salmonella* enterica LPS, *E. coli* O157:H7 outer membrane proteins and shiga toxins, *S. aureus* enterotoxins, and *L. monocytogenes* internalins, highlighting their applicability in foodborne pathogen detection [33].

Aptamers offer several distinct advantages in the field of foodborne pathogens detection. First, they can be produced and screened in vitro, ensuring rapid preparation with minimal batch-to-batch variation [50]. Second, aptamers exhibit superior stability, retaining their structure and binding activity across broader ranges of temperature and pH, which distinguishes them from protein-based receptors [51,52]. Furthermore, their small size and ease of chemical modification allow flexible conjugation with a variety of signal reporters or materials—such as fluorescence probes, electrochemical labels, or Raman tags—significantly enhancing detection sensitivity and operational convenience [53,54,55]. Despite their promise, aptamers still face several challenges in practical applications. Their binding performance may be compromised in real food matrices due to interference from non-specific components, which can reduce sensitivity and specificity [56]. Additionally, aptamers are susceptible to nuclease degradation and non-specific adsorption, leading to unstable detection signals [57]. The range of available aptamers and validated bacterial targets also remains limited, underscoring the need for further exploration of specific binding sites and the development of novel aptamer candidates.

To overcome these limitations, recent optimization strategies include chemical backbone modifications (e.g., 2′-fluoro or locked nucleic acids) to enhance nuclease resistance, structure-guided design to improve conformational stability, and cooperative multi-aptamer approaches that enhance specificity by targeting multiple epitopes simultaneously [58,59,60]. For example, dual-aptamer systems recognizing both surface proteins and toxins of *S. aureus* have demonstrated higher sensitivity than single-aptamer assays, while aptamer–nanomaterial hybrids (e.g., gold nanoparticles, graphene oxide, MOFs) have achieved robust signal amplification in complex food matrices.

Overall, aptamers represent a novel and highly versatile class of recognition elements that are gradually complementing or even replacing traditional antibodies in pathogen detection systems. Future research should focus on advancing aptamer screening technologies, refining structural optimization, and improving robustness in complex food matrices, thereby accelerating their broader implementation in rapid food safety monitoring.

### 3.3. Nucleic Acid

Nucleic acids are essential recognition elements in pathogen detection systems, as their sequence specificity enables precise identification of target microorganisms through complementary base pairing. By designing oligonucleotide probes or primers that selectively hybridize with pathogen-specific genomic regions, nucleic acids provide a highly reliable basis for detection assays [61]. However, because the abundance of target nucleic acids in food or clinical samples is often extremely low, signal amplification methods are typically required to achieve practical sensitivity. Although conventional polymerase chain reaction (PCR) has been widely applied, its dependence on thermal cycling equipment and relatively complex operation limits its suitability for on-site rapid testing [62]. Consequently, isothermal amplification methods have emerged as attractive alternatives for food safety monitoring. The most commonly employed isothermal amplification strategies include loop-mediated isothermal amplification (LAMP), recombinase polymerase amplification (RPA), and strand displacement amplification (SDA) [63,64,65].

LAMP relies on the activity of strand-displacing DNA polymerases and operates at a constant temperature of approximately 60–65 °C [66]. It features exceptional specificity and amplification efficiency, typically producing detectable signals within 30–60 min, with outputs readily visualized by fluorescence, turbidity, or colorimetric methods [67,68,69]. Nonetheless, the requirement for multiple primers increases the risk of nonspecific amplification, and careful primer design remains critical.

RPA has gained increasing attention in recent years. This technique utilizes recombinase, single-stranded binding proteins, and strand-displacing DNA polymerases to mediate rapid amplification at relatively low temperatures (37–42 °C), usually within 20 min [70]. RPA requires only simple primer design, operates efficiently without sophisticated instrumentation, and is highly compatible with portable or microfluidic platforms, making it particularly suitable for field-deployable pathogen testing [71]. However, the extremely high sensitivity of RPA often leads to elevated false-positive rates, highlighting the need to balance sensitivity and specificity [72].

SDA, one of the earlier isothermal amplification strategies, involves the combined action of sequence-specific endonucleases and strand-displacing polymerases at 37 °C [73]. It offers operational simplicity and obviates the need for thermal cycling equipment. Nonetheless, SDA generally exhibits lower amplification efficiency compared with LAMP and RPA, and its reliance on highly specific endonucleases restricts broader application.

Despite the advantages of rapidity, efficiency, and operational simplicity, isothermal amplification still faces several technical barriers in real-world applications. Sample preparation and nucleic acid extraction remain bottlenecks, and issues such as nonspecific amplification and cross-contamination can compromise assay reliability and reproducibility [74,75]. To address these challenges, recent advances have focused on strategies such as optimized primer/probe design, closed-tube reaction systems, and integration with microfluidic chips to improve specificity, reduce contamination risk, and simplify workflows [76,77,78].

Overall, nucleic acid amplification technologies—particularly isothermal methods—have demonstrated tremendous potential in foodborne pathogen detection owing to their speed, sensitivity, and compatibility with portable systems. Future research should prioritize simplification of sample pretreatment, enhancement of amplification specificity, and development of fully integrated portable platforms to facilitate more reliable, user-friendly, and widely deployable solutions for food safety monitoring.

### 3.4. CRISPR/Cas System

The CRISPR-Cas (clustered regularly interspaced short palindromic repeats-CRISPR associated protein) system represents one of the most significant breakthroughs in molecular biology in recent decades. Initially discovered in bacteria and archaea as an adaptive immune mechanism against foreign nucleic acids, it has rapidly been repurposed for nucleic acid detection owing to its highly specific recognition and cleavage capabilities [79]. In recent years, CRISPR-Cas systems have demonstrated tremendous potential in foodborne pathogen detection and are increasingly recognized as a representative class of novel rapid nucleic acid-based diagnostic tools.

The CRISPR-Cas systems commonly applied in detection technologies include Cas9, Cas12, and Cas13. Cas9 primarily mediates recognition and cleavage of specific double-stranded DNA sequences, and while it is more commonly used in genome editing, gene therapy, and crop improvement, Cas12 and Cas13 have become particularly attractive for diagnostics due to their unique “collateral cleavage” activity [80,81,82]. Cas12 recognizes and cleaves double-stranded DNA, whereas Cas13 specifically targets single-stranded RNA [83,84]. Upon recognition of a target nucleic acid, these enzymes engage in non-specific cleavage of reporter probes, producing rapid and highly sensitive signal readouts.

CRISPR-based assays offer several advantages for foodborne pathogen detection. Their extremely high specificity allows single-base discrimination, effectively reducing false positives. Moreover, reactions proceed rapidly, often generating detectable signals within minutes [85]. Within the CRISPR family, Cas12 has been widely applied for the recognition of pathogen-derived DNA targets, while Cas13 demonstrates outstanding performance in detecting RNA biomarkers of pathogens [86,87].

Despite these advantages, several challenges remain. CRISPR assays often require nucleic acid extraction, and complex sample preparation can hinder field-deployable rapid testing [88]. In addition, assay sensitivity and accuracy may be compromised in complex food matrices due to background interference, which can lead to false negatives [89]. To address these limitations, multiple strategies have been developed in recent years. These include rational crRNA (CRISPR RNA) design optimization, development of extraction-free detection workflows, and integration with complementary platforms such as nanomaterials or microfluidic devices to enhance stability and performance [90,91,92,93].

With their outstanding sensitivity, specificity, speed, and portability, CRISPR-Cas systems have become highly promising recognition elements for pathogen detection. Future efforts should focus on enhancing their reliability in complex real-world food samples and advancing their miniaturization, automation, and integration to enable broader, more efficient, and more robust deployment in rapid on-site food safety monitoring.

### 3.5. Molecular Imprinting Technology

Molecular imprinting technology (MIT) is a biomimetic recognition strategy in which template molecules interact with functional monomers to form specific cavities and recognition sites within a polymer matrix, thereby enabling highly efficient and selective capture of target analytes [94]. In recent years, MIT has emerged as a promising recognition element for biosensing due to its excellent tolerance, high stability, low cost, and batch-to-batch reproducibility [95]. These advantages make MIT particularly suitable for rapid detection under harsh environments and within complex food matrices, where it maintains robust recognition performance and reusability [96]. In the field of food safety, especially in pathogen detection, MIT has gradually become a powerful complement to traditional biological recognition elements.

In pathogen detection, MIT typically employs either intact bacterial cells or cell-surface components (e.g., proteins, lipopolysaccharides) as templates. By selecting appropriate functional monomers—such as acrylamide, methacrylic acid, or vinylpyrrolidone—polymers are synthesized with complementary spatial structures that specifically recognize these bacterial features [97]. When combined with transduction modalities such as electrochemistry, fluorescence, or surface plasmon resonance (SPR), MIT has enabled the development of a wide range of rapid biosensors with significantly enhanced sensitivity and detection speed [98,99,100].

Despite its potential, MIT still faces several technical bottlenecks. Traditional imprinting methods often show limited efficiency and recognition capability when applied to large targets such as whole bacterial cells, as effective exposure and accessibility of recognition sites remain challenging [101]. Furthermore, nonspecific adsorption restricts the sensitivity and selectivity of imprinted polymers, especially in complex food matrices where background interference is pronounced [102]. To overcome these limitations, researchers have explored various optimization strategies, including electropolymerization, template surface modification, and surface imprinting technology (SIT) [103,104,105]. These approaches have markedly improved the selective recognition and capture efficiency of MIT toward bacterial targets. For instance, Doostmohammadi et al. demonstrated that tuning the shell thickness of core–shell imprinted microspheres enhanced the imprinting efficiency for *E. coli*, achieving a capture efficiency of 74% compared with 45–65% using conventional methods, highlighting structural optimization as a key route for improving recognition performance [106].

Overall, MIT, with its remarkable stability, cost-effectiveness, and reusability, holds broad potential in foodborne pathogen detection. Future research should focus on enhancing imprinting efficiency and specificity, refining polymer synthesis methods, and promoting integration with advanced detection technologies, thereby improving its performance in complex food samples and accelerating translation into practical food safety applications.

### 3.6. Peptide

Peptides have recently attracted increasing attention as emerging molecular recognition elements in pathogen detection [107]. Composed of short amino acid chains (typically fewer than 50 residues) linked by peptide bonds, peptides feature simple and well-defined structures and can be rapidly obtained through chemical or biological synthesis [108]. Their structural diversity derives from the type, sequence, and arrangement of amino acids, endowing peptides with versatile physicochemical properties and biological activities. This nearly unlimited combinatorial potential makes peptides particularly attractive as bioreceptors. By rationally designing amino acid sequences, peptides can be tailored with the desired hydrophobicity, polarity, length, or rigidity to enhance their selectivity and specificity toward given targets [109,110].

The preparation of peptide recognition elements generally relies on high-throughput screening strategies such as phage display or combinatorial peptide library screening [111,112]. These approaches enable the rapid identification of peptides with high affinity toward specific biomolecules on bacterial surfaces, including membrane proteins, lipopolysaccharides, polysaccharides, or extracellular polymers. Compared with other recognition elements, peptide screening is cost-effective, fast, and amenable to large-scale production. In addition, peptides display superior thermal and chemical stability, maintaining their structural integrity under a wide range of temperatures, pH values, and ionic strengths, thereby ensuring assay reproducibility [113]. Their small size also facilitates chemical modification and functionalization, enabling efficient integration with diverse signal transduction systems—such as fluorescence, electrochemistry, or surface plasmon resonance—to enhance sensitivity and operational simplicity [114,115,116].

Nevertheless, peptides still face several challenges in practical detection applications. Their specificity is often lower than that of antibodies or aptamers, which may lead to nonspecific adsorption or reduced recognition performance in complex food matrices [117]. Moreover, peptides are prone to enzymatic degradation, limiting their stability in certain real-world samples [118]. To address these limitations, a variety of optimization strategies have been proposed, including chemical modification, cyclization, conjugation with nanomaterials, and oriented immobilization. These approaches significantly improve peptide affinity, specificity, and stability [119,120].

In summary, peptides, with their unique advantages and flexible modification potential, are emerging as a promising class of recognition elements for rapid pathogen detection. Future research should focus on advancing peptide screening and design technologies, improving specificity and stability, and integrating peptides with next-generation transduction systems to expand their applicability in food safety monitoring and other real-world scenarios.

### 3.7. Small-Molecule Receptor

Small-molecule receptors are a class of low-molecular-weight compounds (typically <1000 Da) that interact with their targets through noncovalent forces such as hydrogen bonding, electrostatic interactions, hydrophobic interactions, or metal coordination. In the context of pathogen detection, these receptors are often derived from natural antibiotic molecules (e.g., vancomycin, polymyxin) or synthetically engineered functional molecules. Their advantages include low production costs, well-defined chemical structures, high stability, and facile large-scale synthesis, making them attractive recognition elements for sensor design [121].

In practical applications, small-molecule receptors are commonly used to recognize characteristic bacterial cell wall or outer membrane components. A typical example is vancomycin, which specifically binds to the D-Ala-D-Ala moiety in the peptidoglycan of Gram-positive bacteria [122]. Similarly, polymyxin B interacts with the LPS of Gram-negative bacteria, and has been widely exploited in biosensing platforms targeting *E. coli*, *Salmonella*, and other Gram-negative pathogens [123]. However, compared with other recognition elements, small-molecule receptors have pronounced limitations. Because their binding sites are generally conserved cell wall or membrane structures, they are usually restricted to distinguishing between Gram-positive and Gram-negative bacteria, but lack the specificity required to discriminate between bacterial strains [124]. Consequently, small-molecule receptors mainly serve as “broad-spectrum” or “auxiliary” recognition elements and are often combined with more specific elements (e.g., aptamers or antibodies) to form dual-recognition or multiplexed detection platforms. For example, Novakovic et al. developed a portable, low-cost, and highly sensitive dual-recognition electrochemical aptasensor that enabled rapid detection of vancomycin-sensitive Gram-positive bacteria such as *S. aureus* and *Bacillus cereus* (*B. cereus*) in complex food and clinical samples, demonstrating promising applications in food safety, clinical diagnostics, and environmental monitoring [125].

Future research directions may include structural modification of small molecules, integration with nanomaterials, and coupling with advanced signal amplification strategies to further enhance their utility in rapid foodborne pathogen detection.

In summary, recognition elements such as antibodies, aptamers, nucleic acid amplification technologies, CRISPR/Cas systems, molecular imprinting polymers, peptides, and small-molecule receptors each present unique strengths and limitations for foodborne pathogen detection. They differ substantially in terms of specificity, sensitivity, and applicability. To provide a systematic comparison, Table 1 summarizes the key performance characteristics of these seven recognition elements.

## 4. Detection Methods and Innovative Rapid Strategies

In the preceding sections, we systematically reviewed various biological recognition elements and discussed their mechanisms, advantages, and limitations in foodborne pathogen detection. However, recognition elements alone provide specificity and selectivity; the overall detection performance is largely determined by the associated signal transduction methods and detection platforms. In recent years, driven by rapid advances in materials science, microfluidics, and information engineering, a variety of innovative rapid detection strategies have emerged. These include lateral flow assays, electrochemical biosensors, optical and fluorescence sensors, as well as Raman- and surface-enhanced Raman scattering (SERS)-based approaches. Collectively, these strategies not only offer substantial advantages in sensitivity, response speed, and operational simplicity but also create new opportunities for multiplex detection, point-of-care testing (POCT), and intelligent applications.

### 4.1. Conventional Rapid Methods

#### 4.1.1. Lateral Flow Assays

Lateral flow assays (LFAs) are a class of rapid diagnostic technologies driven by capillary action, enabling liquid samples to migrate along test strips while carrying out recognition and signal transduction [126]. The principle involves adding the sample to a loading pad, after which the liquid migrates through the nitrocellulose membrane. If the target analyte is present, it binds to recognition elements immobilized at the test line and triggers a visible signal. Because LFAs require no complex instrumentation, are simple to operate, and deliver rapid results, they have been extensively applied in the on-site screening of foodborne pathogens.

Structurally, a typical LFA consists of a sample pad, a conjugate pad, a nitrocellulose membrane, and an absorbent pad. The sensitivity and clarity of signal readout are largely determined by the properties of the labeling particles. Gold nanoparticles (AuNPs), owing to their unique surface plasmon resonance effect, are the most widely used and well-established signal reporters, producing easily visible red test lines. In recent years, novel probes such as quantum dots, fluorescent microspheres, upconversion nanoparticles, and enzyme labels have been introduced, significantly enhancing assay sensitivity and enabling the transition of LFAs from qualitative outputs (“yes/no” results) to quantitative and multiplex detection capabilities [127].

**Figure 3:**
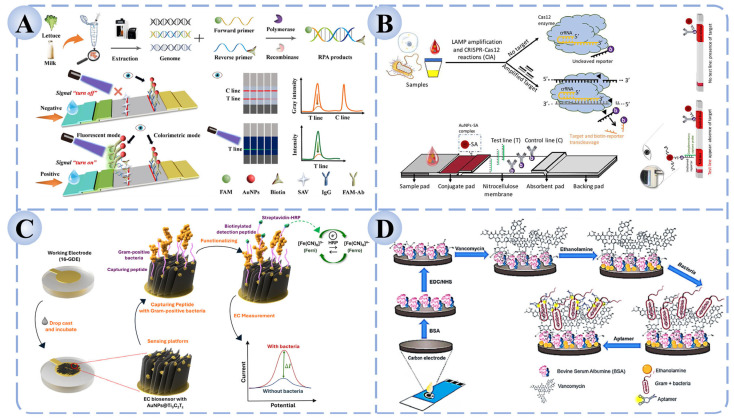
LFA and Electrochemical biosensors for foodborne pathogen detection based on recognition elements. (**A**) Dual-mode (colorimetric/fluorescent) RPA-LFA strip for *Salmonella* detection [128]. (**B**) CRISPR/Cas12 coupled with LAMP-based CIA-LFB for the detection of *P. aeruginosa* [129]. (**C**) Electrochemical biosensor based on AuNPs@Ti_3_C_2_T_Z_ functionalized sandwich peptides [130]. (**D**) Dual-recognition electrochemical sensor combining vancomycin and aptamers for bacterial detection [125].

The specificity of LFAs depends primarily on the recognition element employed. Early LFAs largely relied on antibodies as the central binding molecules; however, limitations in antibody stability and batch-to-batch variation restricted their application scope. With further research, aptamers, nucleic acid probes, and CRISPR-based systems have been integrated into LFA platforms [15]. For example, as illustrated in Figure 3A, Feng et al. developed an RPA-based dual-mode (colorimetric/fluorescent) LFA that enabled ultrahigh-sensitivity detection of *Salmonella* in milk and lettuce within 10 min, with detection limits of 10 CFU/mL (colorimetric) and 1 CFU/mL (fluorescent), demonstrating high accuracy and robustness in real food samples [128]. Similarly, Mukama et al. constructed a CRISPR/Cas12a-assisted LAMP–lateral flow biosensor (CIA-LFB) for the low-cost, rapid, and ultrasensitive visual detection of *Pseudomonas aeruginosa* (*P. aeruginosa*), showing great promise for clinical and field applications (Figure 3B) [129].

In summary, LFAs, as a mature yet continuously evolving paper-based detection method, are breaking through traditional limitations in sensitivity and quantification by integrating novel recognition elements, signal amplification strategies, and portable readout devices. These innovations position LFAs as powerful tools for on-site and large-scale screening of foodborne pathogens.

#### 4.1.2. Electrochemical Biosensors

Electrochemical biosensors represent a class of analytical tools that transduce biological recognition events into electrochemical signals. Their core principle relies on the fact that when recognition elements bind to target pathogens or specific biomarkers, measurable changes occur at the electrode interface, such as charge transfer, impedance, or current responses, thereby enabling quantifiable detection [131]. Common electrochemical techniques include differential pulse voltammetry (DPV), cyclic voltammetry (CV), square wave voltammetry (SWV), and electrochemical impedance spectroscopy (EIS), all of which sensitively reflect interfacial changes induced by biomolecular interactions. These methods provide unique advantages for the detection of pathogens at low concentrations.

To overcome limitations in sensitivity and stability, researchers have increasingly introduced novel functional materials and interface modification strategies. Nanomaterials such as carbon nanotubes, graphene, gold nanoparticles, and metal–organic frameworks (MOFs) are widely employed owing to their high specific surface area, excellent conductivity, and facile surface functionalization. These materials not only enhance electron transfer efficiency but also provide abundant immobilization sites for recognition molecules, effectively amplifying detection signals [132]. By combining these functional interfaces with specific recognition elements, electrochemical biosensors are evolving toward greater selectivity and broader application potential.

Recent studies demonstrate that the synergistic integration of nanomaterials with recognition elements can significantly extend the utility of electrochemical detection. For example, as illustrated in Figure 3C, Park et al. designed a high-throughput electrochemical detection platform utilizing AuNPs@Ti_3_C_2_T_Z_-functionalized sandwich peptides. Through the synergistic effect of specific peptide recognition and the high conductivity and signal amplification provided by nanomaterials, the system achieved ultrasensitive detection of *S. aureus*, *B. cereus*, and *Micrococcus luteus*, with detection limits as low as 8–15 CFU/mL—representing a 1–2 order of magnitude improvement over traditional electrochemical methods [130]. Similarly, Novaković et al. developed a portable, low-cost, and highly sensitive dual-recognition electrochemical aptasensor capable of rapidly detecting and identifying vancomycin-sensitive Gram-positive bacteria (such as *S. aureus* and *B. cereus*) in complex food and clinical samples. This platform demonstrates broad potential for applications in food safety, clinical diagnostics, and environmental monitoring (Figure 3D) [125].

Overall, electrochemical biosensors, through the deep integration of functional materials and recognition elements, are gradually transitioning from single-target detection toward high sensitivity, multiplexing, and on-site applicability. These advances lay a solid foundation for the development of efficient and portable detection platforms for foodborne pathogens in the future.

#### 4.1.3. Fluorescence Biosensors

Fluorescence biosensors are a class of detection tools that achieve target recognition through fluorescence signal transduction. Their core mechanism is based on changes in fluorescence emission or quenching that occur when recognition elements bind to the target, enabling both qualitative and quantitative analysis [133]. Compared with conventional culture-based methods, fluorescence sensing offers distinct advantages such as rapid response, high sensitivity, and intuitive readout, making it particularly suitable for the rapid screening of pathogens at low concentrations. For signal acquisition, fluorescence biosensors typically rely on fluorescence microscopy, spectrophotometers, or portable fluorescence detectors, while the recent integration of smartphones and miniaturized optoelectronic modules has further accelerated their application in POCT.

**Figure 4:**
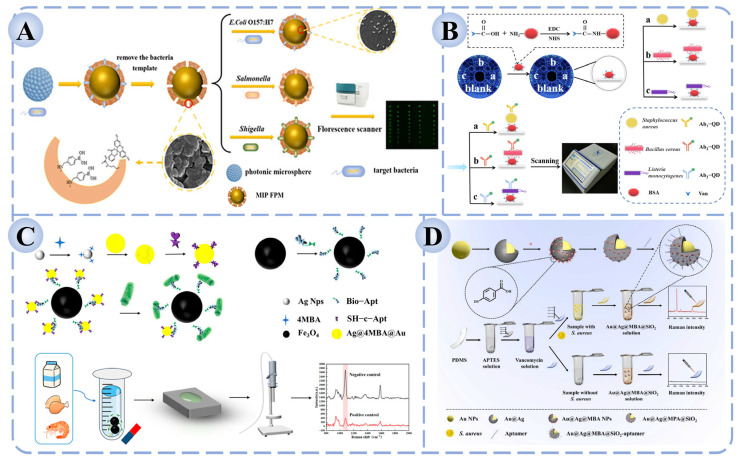
Fluorescence and Raman biosensors for foodborne pathogen detection based on recognition elements. (**A**) Fluorescence immunosensor based on dual recognition of vancomycin and antibodies [135]. (**B**) High-throughput fluorescence sensor based on molecular imprinting for bacterial detection [136]. (**C**) Aptamer-functionalized Fe_3_O_4_ magnetic bead–Ag@Au SERS sensor for *Salmonella* detection [137]. (**D**) Sandwich-type SERS biosensor for the detection of *S. aureus* [138].

In sensor construction, functional materials play a crucial role in amplifying and stabilizing fluorescence signals. Quantum dots, fluorescent nanoparticles, rare-earth-doped nanomaterials, and carbon dots have been widely employed as signal carriers due to their high brightness, photostability, and facile surface modification [134]. These materials provide an efficient platform for signal transduction, while the incorporation of recognition elements imparts high specificity. For example, antibody-based fluorescent immunosensors often exhibit superior sensitivity and rapid response. Cheng et al. developed a dual-recognition fluorescence immunoassay based on vancomycin and antibodies on a microfluidic chip, enabling the simultaneous and sensitive detection of *S. aureus*, *B. cereus*, and *L. monocytogenes*, with successful validation in milk samples (Figure 4A) [135]. Similarly, as shown in Figure 4B, Zhang et al. constructed a whole-cell molecularly imprinted photonic crystal microsphere array platform, which allowed high-throughput and ultrasensitive detection of *Salmonella*, *Shigella*, and *E. coli* O157:H7 without the need for molecular probes or amplification steps [136].

Overall, fluorescence biosensors are evolving toward higher sensitivity, multiplexing capability, and portability. By integrating novel functional materials with microfluidic systems, fluorescence sensing shows great promise for applications in food safety monitoring and public health surveillance.

#### 4.1.4. Raman Biosensors

Raman biosensors represent an analytical approach for pathogen detection based on the Raman scattering effect, in which the frequency shift of scattered light reveals molecular structural information. Because of their fingerprint-like spectral characteristics, Raman spectroscopy offers unique molecular markers for the rapid identification of foodborne pathogens [139]. However, conventional Raman signals are inherently weak, limiting their applicability in trace pathogen detection. To overcome this limitation, surface-enhanced Raman spectroscopy (SERS) has been developed. By leveraging the strong electromagnetic fields and chemical enhancement effects generated by noble metal nanoparticles or nanostructured substrates, SERS amplifies Raman signals by several orders of magnitude, thereby enabling the detection of single bacteria or ultralow concentrations of biomarkers [140].

The design of nanostructured substrates is crucial for constructing effective SERS platforms. Gold (Au) and silver (Ag) nanoparticles are the most commonly used due to their outstanding surface plasmon resonance effects, while morphology-controlled structures—such as core–shell nanostructures, nanoflowers, and nanorods—further enhance electromagnetic field intensity and significantly improve signal reproducibility. In recent years, multifunctional substrates composed of composites of plasmonic nanoparticles with metal–organic frameworks (MOFs) or carbon-based nanomaterials have been increasingly introduced, aiming to enhance detection sensitivity while reducing background interference [141]. Regarding the integration of recognition elements, SERS biosensors achieve high selectivity by combining with antibodies, aptamers, or small-molecule ligands to capture target pathogens. For instance, as shown in Figure 4C, Sun et al. developed an aptamer-functionalized Fe_3_O_4_ magnetic bead–Ag@Au SERS biosensor, which enabled accurate quantification of five *Salmonella* strains within the range of 10^2^–10^8^ CFU/mL, with a detection limit of 35.51 CFU/mL and recovery rates of 94.0–100.4% in milk, chicken, and shrimp samples [137]. Similarly, Wei et al. reported a sandwich-type SERS biosensor for *S. aureus* detection, in which vancomycin was immobilized on PDMS (Polydimethylsiloxane) films as the capture molecule and aptamer-modified Au@Ag@SiO_2_ nanoprobes served as the signal unit (Figure 4D) [138]. In the presence of the target bacterium, the capture and signal units assembled into a sandwich structure on the bacterial surface, generating a stable and strong Raman signal. This approach achieved a detection limit as low as 2 CFU/mL, with excellent selectivity and recovery performance in real food samples such as fish and milk, thereby highlighting its potential in food safety monitoring.

To provide a more intuitive comparison of the performance differences among various detection strategies, Table 2 summarizes the key features and performance metrics of foodborne pathogen detection methods based on different recognition elements.

### 4.2. Integration and Multianalyte Platforms

In practical food safety testing, contamination often involves multiple pathogenic species and complex sample matrices, where single-target or single-recognition approaches are insufficient to achieve comprehensive coverage. As a result, multi-target and multimodal integrated platforms have emerged as a major research focus [155,156]. By integrating multiple recognition elements or combining diverse signal transduction pathways, such platforms are capable of simultaneously identifying multiple pathogens and collecting multisource signals, thereby enhancing accuracy, throughput, and resistance to interference.

**Figure 5:**
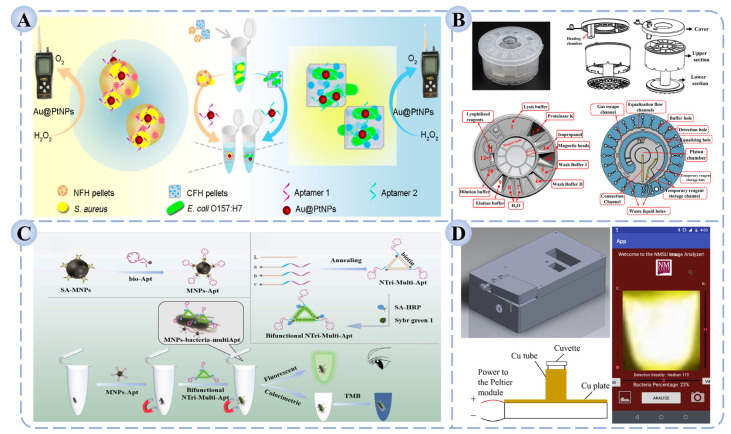
Schematic illustration of (**A**) a detection platform integrating shape-encoded hydrogel particles with a pneumatic sensor for the simultaneous detection of *S. aureus* and *E. coli* O157:H7 [159]; (**B**) a dual-mode colorimetric/fluorescent detection platform based on DNA nanotrimer aptamers and magnetic separation for the detection of *Salmonella* [163]; (**C**) a fully enclosed microfluidic cartridge for automated pathogen detection [164]; (**D**) a portable smartphone-based LAMP platform for the visual detection of *S. aureus* [165].

Multi-target platforms often rely on spatial partitioning or encoding probes to enable the synchronous detection of different pathogens [157,158]. For instance, as shown in Figure 5A, Li et al. developed a detection platform based on shape-encoded hydrogel particles coupled with a pneumatic sensor [159]. By loading distinct hydrogel particles with Au@Pt core–shell nanoparticles modified with pathogen-specific aptamers, the platform achieved multiplex detection of *S. aureus* and *E. coli* O157:H7 with limits of detection as low as 1.4 × 10^3^ CFU/mL and 5.3 × 10^2^ CFU/mL, respectively, while maintaining high recovery rates and specificity in complex food samples. Such designs are particularly advantageous for on-site emergency testing and high-throughput food screening, significantly improving operational efficiency and risk-response capacity.

Multimodal platforms, by contrast, emphasize the integration of different signal transduction and analytical modalities. For example, combining fluorescence with electrochemical or colorimetric readouts enables a “mutual verification” mechanism that enhances result reliability [160,161]. Furthermore, multimodal systems help compensate for the limitations of single technologies in terms of sensitivity or background interference, making them more broadly applicable across complex food matrices [162]. Ma et al. constructed a dual-mode colorimetric/fluorescent platform based on DNA nanotriangle multivalent aptamers and magnetic separation, where the two signal modes served as cross-validation [163]. This strategy effectively reduced false positives and false negatives, and in multiple complex food samples, the coefficient of variation for *Salmonella* detection remained below 9.53%, indicating excellent accuracy (Figure 5B).

Despite their remarkable performance, multi-target and multimodal platforms face challenges such as complex structural design, stringent requirements for reaction coordination, and the need for advanced data processing, which collectively raise concerns about integration stability and operational simplicity [166]. Future research will likely prioritize the development of higher-throughput, lower-cost, and more modular integrated detection systems, while promoting their convergence with automation, microfluidics, and intelligent algorithms. Such advancements are expected to enable truly unified “multi-target–multimodal–integrated” rapid detection platforms for foodborne pathogens.

### 4.3. Microfluidics and Portable POCT Devices

To meet the demands of on-site rapid testing and applications in resource-limited settings, POCT has attracted increasing attention in pathogen detection in recent years [167]. POCT emphasizes “portability, efficiency, and ease of operation,” with the core goal of enabling non-specialists to complete sample testing and obtain results directly at the point of need. This approach offers unique advantages in food safety monitoring, environmental surveillance, and emergency responses to public health incidents, providing rapid reaction and large-scale screening capabilities.

Microfluidics, as a key technological foundation of POCT, precisely manipulates liquid flow in micron- to nanometer-scale channels, enabling the integration of complex processes such as sample pretreatment, target recognition, signal amplification, and result readout within a single chip [168,169]. Compared with conventional laboratory-based assays, microfluidic chips substantially reduce the consumption of samples and reagents, while offering rapid reaction kinetics, multi-step automation, and high-throughput parallel testing [170,171]. In bacterial detection, microfluidic platforms can integrate sample lysis, nucleic acid extraction, amplification, and detection into a single streamlined workflow, thereby minimizing cross-contamination and human error [172]. As illustrated in Figure 5C, Tang et al. developed a fully enclosed microfluidic cartridge-based automated system that integrates nucleic acid extraction, lyophilized reagent preparation, LAMP amplification, and signal detection [164]. The device simultaneously detected *S. aureus*, *L. monocytogenes*, *Vibrio parahaemolyticus*, and *Salmonella typhimurium* within 45 min, achieving a sensitivity of 500 CFU/mL. Similarly, Yin et al. constructed a portable fluorescence biosensor based on a 3D-printed microfluidic chip coupled with smartphone imaging [173]. By integrating magnetic molecularly imprinted materials with aggregation-induced emission (AIE) fluorescent probes, the platform enabled multiplex detection of *E. coli*, *S. aureus*, and *P. aeruginosa*. The assay quantified results through RGB analysis of smartphone-acquired images, achieved detection limits as low as 10^2^ CFU/mL within 40 min, and demonstrated high recovery rates and stability in milk, water, and juice samples. Such “microfluidic–POCT hybrid platforms” provide low-cost, rapid, and scalable solutions for food safety monitoring and emergency on-site testing.

Despite their promise, microfluidic and POCT systems face several challenges in practical deployment. These include the high precision required for fluidic control, limited compatibility among functional modules, elevated manufacturing costs, and insufficient long-term storage stability. Furthermore, performance often declines in complex food matrices, where reduced sensitivity or false negatives may occur. To overcome these barriers, future research should prioritize the use of low-cost materials and modular fabrication strategies, surface modification approaches to enhance stability and antifouling properties, and standardized designs to simplify workflows. In addition, stronger integration with smart terminals and cloud-based data platforms will be crucial for enabling real-time result display, cloud analysis, and remote data sharing. With the further convergence of AI and IoT, microfluidic–POCT platforms hold great potential to establish an “intelligent food safety monitoring network,” facilitating the transition of pathogen detection from on-site applications toward large-scale industrialization.

### 4.4. Intelligent Terminals and Artificial Intelligence-Assisted Detection

With the rapid advancement of information technologies such as IoT, AI, and mobile computing, pathogen detection technologies are entering a new era of intelligence and digitalization [174,175]. Integrating conventional detection systems with smart terminals and AI algorithms enables automated data acquisition, image analysis, pattern recognition, and result prediction, thereby providing more powerful technical support and decision-making capacity for on-site rapid testing [176,177,178].

Smart terminals play critical roles in pathogen detection, including signal acquisition and interpretation, data analysis, visualization of results, and remote data sharing [179,180,181]. As illustrated in Figure 5D, Cabrales-Arellano et al. developed a portable platform integrating LAMP with smartphone-based analysis [165]. In this system, the smartphone conducted signal collection, data processing, and visualization, enabling the sensitive detection of *S. aureus* within one hour with a detection limit of 10^3^ CFU/mL. Such approaches markedly enhance testing convenience and are particularly suitable for applications in remote regions or resource-limited environments.

**Figure 6:**
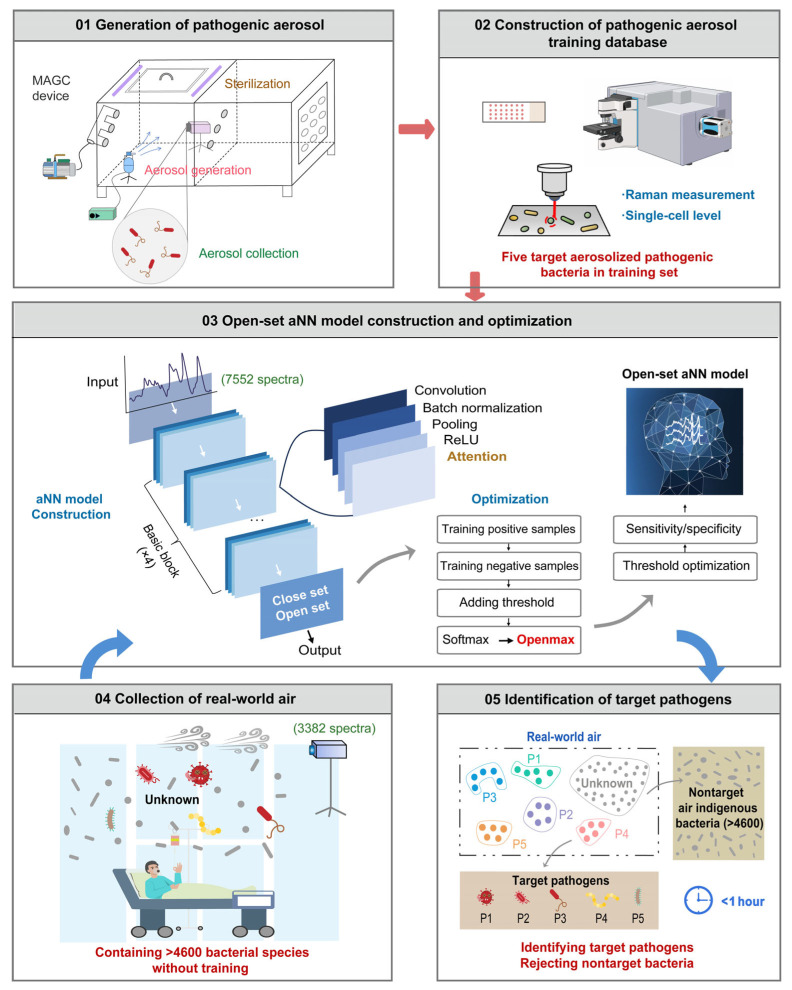
Schematic illustration of a workflow for rapid aerosolized pathogen identification using single-cell Raman spectroscopy combined with open-set deep learning [184].

AI-assisted detection focuses on employing machine learning and deep learning algorithms for intelligent data processing, such as image classification, signal recognition, anomaly detection, and multi-target pattern matching [182,183]. For instance, Zhu et al. proposed a strategy combining single-cell Raman spectroscopy with an open-set deep learning (OSDL) model, establishing a Raman spectral database of aerosolized pathogens and optimizing the algorithm to enable rapid, culture-free identification of multiple pathogens in real air samples [184]. The method achieved 93% accuracy, reduced false positives by 36%, and completed testing within approximately one hour (Figure 6). Furthermore, leveraging cloud platforms and IoT connectivity allows detection devices to interact with cloud servers for remote monitoring, big data analysis, and trend forecasting, providing digitalized support for food supply chain safety management [185].

Despite their promise, smart terminal- and AI-assisted detection systems still face challenges in large-scale implementation, including algorithm reliability, model generalizability, hardware compatibility, and data privacy protection. Future efforts should focus on the synergistic optimization of “algorithm–platform–application”, emphasizing lightweight AI models, localized deployment, and seamless adaptation to portable devices. Ultimately, this will foster the development of truly integrated intelligent detection systems with capabilities spanning “detection–analysis–prediction”.

In summary, this chapter outlines the emerging trends in foodborne pathogen detection, emphasizing multi-target and multimodal platforms, microfluidics-enabled POCT systems, and intelligent detection terminals powered by AI. Multi-target and multimodal platforms improve parallelism and reliability of results; microfluidic–POCT devices significantly enhance portability and on-site applicability; while smart terminals and AI integration drive the field toward digitalization and intelligence. Table 3 summarizes representative studies and performance advantages of these platforms, highlighting significant progress in sensitivity, throughput, and application scope. Nevertheless, challenges such as cost, stability, and model generalization remain, and future research will focus on modular and intelligent integration to accelerate the translation of laboratory innovations into scalable real-world applications.

## 5. Discussion

This review centers on the application of recognition elements in the rapid detection of foodborne pathogens, systematically summarizing the research progress of seven core tools: antibodies, aptamers, nucleic acids, CRISPR/Cas systems, molecularly imprinted polymers, peptides, and small-molecule receptors. As the fundamental components of detection systems, these recognition elements determine the specificity, sensitivity, and applicability of the methodologies. In recent years, with advances in molecular engineering, materials science, and bioinformatics, recognition elements have continuously expanded their target scope, improved affinity and stability, and progressively achieved deep integration with multimodal detection technologies. Through coupling with lateral flow assays, electrochemical, fluorescence, and Raman-based sensing platforms, recognition elements have provided a robust foundation for multi-target detection, portable devices, and intelligent analysis. Looking forward, the continued optimization of recognition elements and their convergence with emerging technologies such as microfluidics, artificial intelligence, and the Internet of Things are expected to drive pathogen detection toward higher sensitivity, specificity, and accessibility, thereby playing an increasingly pivotal role in food safety and public health.

## Figures and Tables

**Table 1:** Comparison of seven types of recognition elements for foodborne pathogen detection.

Recognition Element	Affinity (Kd)	Specificity	Stability	Cost	Application Scenarios	Limitations
Antibody	nM–pM	High, specific to antigens such as LPS and proteins	Sensitive to temperature/pH	High	Immunochromatography, ELISA, immunofluorescence	Long preparation cycle, high cost, risk of cross-reactivity
Aptamer	nM–pM	High, targeting proteins, toxins, and nucleic acids	Stable under diverse conditions	Medium	Fluorescence, electrochemical, SERS biosensors	Matrix interference, risk of nuclease degradation
Nucleic Acid	fM–nM (with amplification)	Very high, based on complementary base pairing	Moderate, susceptible to degradation	Medium	PCR, LAMP, RPA and other amplification techniques	Complex sample pretreatment, false-positive risk
CRISPR/Cas System	aM–fM	Extremely high, single-base discrimination	Moderate, depends on nucleic acid extraction	Medium–High	Rapid nucleic acid detection, POCT	Complex sample preparation, system optimization required
Molecular Imprinting Technology	μM–nM	Moderate, depending on template molecules	High, resistant to heat, acid, and alkali	Low	Electrochemical, optical, SPR detection	Limited efficiency for large biomolecules, nonspecific adsorption
Peptide	μM–nM	Moderate, can be improved by sequence design	High, robust under diverse conditions	Low	Electrochemical, fluorescence, biochips	Lower specificity, enzymatic degradation risk
Small-Molecule Receptor	μM–nM	Relatively low, broad-spectrum recognition	High, chemically stable	Low	Antibiotic-derivative detection, auxiliary recognition	Limited strain-level discrimination, insufficient specificity

**Table 2:** Comparison of foodborne pathogen detection methods based on different recognition elements and their performance characteristics.

Recognition Elements	Target Analyte	Detection Method	Limit of Detection	Linear Range	Detection Time	Real Sample	Recovery Rate	Reference
Antibody	Vibrio parahaemolyticus	Raman	1 CFU/mL	3–2.2 × 10^8^ CFU/mL	180 min	Water	95–107%	[142]
	*E. coli*	Electrochemistry	0.1 CFU/mL	0.1–10^5^ CFU/mL	10 min	Water	94–109%	[143]
	*E. coli* O157:H7	Fluorescence	7 CFU/mL	0–10^6^ CFU/mL	120 min	Milk	87.10–109.82%	[144]
Aptamer	*Salmonella*	Raman	35.51 CFU/mL	10^2^–10^8^ CFU/mL	120 min	Milk, chicken, shrimp	94–100.4%	[140]
	*S. aureus*	Fluorescence	6 CFU/mL	36–3.6 × 10^7^ CFU/mL	110 min	Water	96–105%	[145]
						Milk	93–106%	
						Tea	88–101%	
						Fish	92–95%	
	*S. aureus*	Fluorescence	25 CFU/mL	63–6.3 × 10^6^ CFU/mL	30 min	Pork	91–93%	[146]
						Beef	96–105%	
Nucleic acid	*S. aureus*	LFA	10^7^ CFU/mL		120 min	Blood		[147]
	*Salmonella typhimurium*	LFA+ Fluorescence	0.1 CFU/mL	10^2^–10^7^ CFU/mL	15 min	Water, juice, lettuce, chicken	85–110%	[148]
	*Salmonella*	LFA	1 CFU/mL	10^−1^–10^4^ CFU/mL	30 min	Milk		[149]
CRISPR/Cas	*P. aeruginosa*	LFA	1 CFU/mL	1–10^8^ CFU/mL	35 min	Milk		[131]
	*S. aureus*	Electrochemistry	3 CFU/mL	1.06–1.06 × 10^8^ CFU/mL	30 min	Milk		[150]
	*S. aureus*	Fluorescence	3 CFU/mL	7.9–7.9 × 10^8^ CFU/mL	80 min	Egg	98.77–104.13%	[85]
MIT	*Salmonella typhimurium*	Fluorescence	17.38 CFU/mL	10–10^7^ CFU/mL	136 min	Chicken	94.7–102.1%	[99]
	*S. aureus*	Electrochemistry	1 CFU/mL	10–10^7^ CFU/mL	110 min	Milk	96–104%	[151]
	*S. aureus*	Fluorescence	11.12 CFU/mL	10–10^7^ CFU/mL			97.7–101.9%	[152]
Peptide	*Vibrio parahaemolyticus*	Electrochemistry	4 CFU/mL	10–10^7^ CFU/mL	30 min	Shrimp	97–106%	[153]
	*E. coli* O157:H7	Fluorescence	2 CFU/mL	5–5 × 10^6^ CFU/mL	50 min	Pork, cabbage, milk		[154]
	*S. aureus*	Electrochemistry	50 CFU/mL	50–10^6^ CFU/mL	170 min	Water		[132]
Small-molecule receptor	*S. aureus*	Electrochemistry	2.7 CFU/mL	10^2^–10^9^ CFU/mL	10 min	Milk		[125]
	*S. aureus*	Raman	2 CFU/mL	38–3.8 × 10^7^ CFU/mL	95 min	Fish	90.51–97.97%	[143]
						Milk	91.32–98.38%	

**Table 3:** Research progress on multi-target, multimodal, POCT, and smart terminal platforms for the detection of foodborne pathogens.

Platform Type	Recognition Elements	Target Analyte	Technical Features	Performance	Advantages	Real Sample	Reference
Multi-target platform	MIT	*Salmonella*	3D photonic microsphere microarray	3 CFU/mL	high-throughput and low-cost detection	Water, milk	[135]
		*Shigella*		20 CFU/mL			
		*E. coli*		1 CFU/mL			
	Peptide	*L. monocytogenes*	Electrode array	9 CFU/mL	High-throughput and low-cost detection		[115]
		*S. aureus*		3 CFU/mL			
	Aptamer	*S. aureus*	Shape-encoded functional hydrogel pellets	1.4 × 10^3^ CFU/mL	A facile, cost-effective, and portable gas pressure sensor	Water	[159]
		*E. coli*		5.3 × 10^2^ CFU/mL			
Multimodal platform	Aptamer	*Vibrio parahaemolyticus*	Colorimetry	9 CFU/mL	Signal cross-validation, anti-interference	Shrimp	[186]
			Raman	7 CFU/mL			
	Aptamer	*S. aureus*	Fluorescence	22 CFU/mL	Signal cross-validation, anti-interference	Pork, beef	[187]
			Colorimetry	20 CFU/mL			
	Aptamer	*Salmonella*	Fluorescence	60 CFU/mL	Signal cross-validation, anti-interference	Milk, egg, chicken	[163]
			Colorimetry	316 CFU/mL			
POCT	Nucleic Acid	*Vibrio parahaemolyticus*, *Salmonella typhimurium*, *L. monocytogenes*, *S. aureus*	Fully enclosed chip	500 CFU/mL, 45 min	Automation, rapid detection	Meat products, egg products, aquatic products	[173]
	Aptamer	*E. coli*, *S. aureus*, *P. aeruginosa*	3D-printed microfluidic chip combined with smartphone	100 CFU/mL, 40 min	Portable and low-cost detection	Water, orange juice, milk	[158]
	Antibody	*S. aureus*	A multifunction and portable 3D-printed pretreatment device	100 CFU/mL, 180 min	Highly sensitive and graded detection	Solid and semi-solid food samples	[188]
Smart terminal platform	Aptamer	*S. aureus*	Enhance the portability of detection with smartphone app	6.9 CFU/mL, 50 min	Suitable for areas with limited resources	Milk	[189]
	Nucleic Acid	*S. aureus*	Smartphone-based signal acquisition and visualization	900 CFU/mL, 60 min	Real-time analysis	Milk	[165]
		Multiple foodborne pathogens	Raman spectroscopy combined with deep learning	Accuracy of 93%	Rapid, culture-free identification	Air	[183]

## Data Availability

No new data were created or analyzed in this study.

## Abbreviations

The following abbreviations are used in this manuscript:

*S. aureus*	*Staphylococcus aureus*
*E. coli*	*Escherichia coli*
*L. monocytogenes*	*Listeria monocytogenes*
WHO	World Health Organization
AI	artificial intelligence
IoT	Internet of Things
ELISA	enzyme-linked immunosorbent assay
LPS	lipopolysaccharides
pAbs	polyclonal antibodies
mAbs	monoclonal antibodies
SPA	staphylococcal protein A
SELEX	systematic evolution of ligands by exponential enrichment
SERS	surface-enhanced Raman scattering
BDS	bridge DNA synthesis
SPANI	sulfonated polyaniline
PCR	polymerase chain reaction
LAMP	loop-mediated isothermal amplification
RPA	recombinase polymerase amplification
SDA	strand displacement amplification
CRISPR	clustered regularly interspaced short palindromic repeats
Cas	CRISPR associated protein
GO	graphene oxide
crRNA	CRISPR RNA
MIT	molecular imprinting technology
SPR	surface plasmon resonance
SIT	surface imprinting technology
*B*. *cereus*	*Bacillus cereus*
POCT	point-of-care testing
LFAs	lateral flow assays
AuNPs	gold nanoparticles
CIA-LFB	CRISPR/Cas12a-assisted LAMP–lateral flow biosensor
*P*. *aeruginosa*	*Pseudomonas aeruginosa*
DPV	differential pulse voltammetry
CV	cyclic voltammetry
SWV	square wave voltammetry
EIS	electrochemical impedance spectroscopy
MOFs	metal–organic frameworks
PDMS	polydimethylsiloxane
AIE	aggregation-induced emission
OSDL	open-set deep learning
